# PYCR2 Mutation Causing Hypomyelination and Microcephaly in an Indian Child

**DOI:** 10.7759/cureus.14661

**Published:** 2021-04-24

**Authors:** Preeti Srivastava, Asit Kumar Mishra, Nilanjan Sarkar

**Affiliations:** 1 Pediatrics, Tata Main Hospital, Jamshedpur, IND; 2 Radiology, Tata Main Hospital, Jamshedpur, IND

**Keywords:** microcephaly, hypomyelination, developmental delay, pycr2 mutation, failure to thrive

## Abstract

Hypomyelinating leukodystrophy (HLD) represents a group of clinically overlapping but genetically heterogeneous diseases. This group of disorders has the improper formation of myelin sheaths in the central nervous system (CNS), resulting in abnormal white matter, with characteristic MRI findings and clinical presentations of mostly motor dysfunction with variable cognitive and language impairment.

We report a case of a three-year-old boy with global developmental delay, dysmorphic facies, motor signs, progressive microcephaly, and failure to thrive. The child was born of a non-consanguineous marriage. All basic investigations and metabolic tests were normal. Magnetic resonance imaging (MRI) of the brain showed hypomyelination of the deep and subcortical white matter, appearing as hyperintense T2 and isointense T1-weighted images, cerebral atrophy with the thinning of the corpus callosum, with normal cerebellum, brainstem, and deep grey nuclei. Further genetic testing in the form of clinical exome sequencing revealed compound heterozygous mutation of the PYCR2 gene and matching the clinical phenotype with the genotype. Therefore, a final diagnosis of hypomyelinating leukodystrophy-10 was made.

There is a wide range of aetiologies for debilitating neurologic disorders, which have common and overlapping clinical presentations. Advances in the field of genetics, growing awareness, and availability of genetic tests help in a better workup of complex neurological cases. A precise diagnosis is useful in outlining the course, treatment (if available), and prognosis of the disease to parents and plays a vital role in planning future pregnancies.

## Introduction

Hypomyelinating leukodystrophy (HLD) represents a group of clinically overlapping but genetically heterogeneous diseases [1]. These patients have an inadequate formation of myelin sheaths in the central nervous system (CNS), resulting in abnormal white matter with characteristic magnetic resonance imaging (MRI) findings and clinical presentations of mostly motor dysfunction with variable cognitive and language impairment [2]. Microcephaly is defined as a head circumference less than 3 SD or Z score < -3. Microcephaly may be observed in 0.1% of the general asymptomatic population but its prevalence is 15%‑20% in children with developmental delay [3]. The etiologic spectrum of microcephaly can be wide-ranging from perinatal hypoxic-ischemic injuries, in-utero infections, post-infective sequelae, and structural abnormalities to less common causes like hypomyelinating, metabolic or genetic disorders. Genetic mutations causing these hypomyelination syndromes are being increasingly recognized. Pelizaeus-Merzbacher disease (PMD) due to proteolipid protein 1 (PLP1) gene mutation and hypomyelinating leukodystrophy-6 due to the tubulin beta-4A gene (TUBB4A) mutation are better known genetic syndromes causing hypomyelination and microcephaly. Recently, Nakayama et al., in 2015, for the first time, identified a mutation in the PYCR2 gene causing a similar phenotypic and radiologic presentation [4]. Here, we report a rare case of hypomyelination, microcephaly, reduced white matter volume, and identification of a mutation in the PYCR2 gene in an Indian child.

## Case presentation

**Figure 1:**
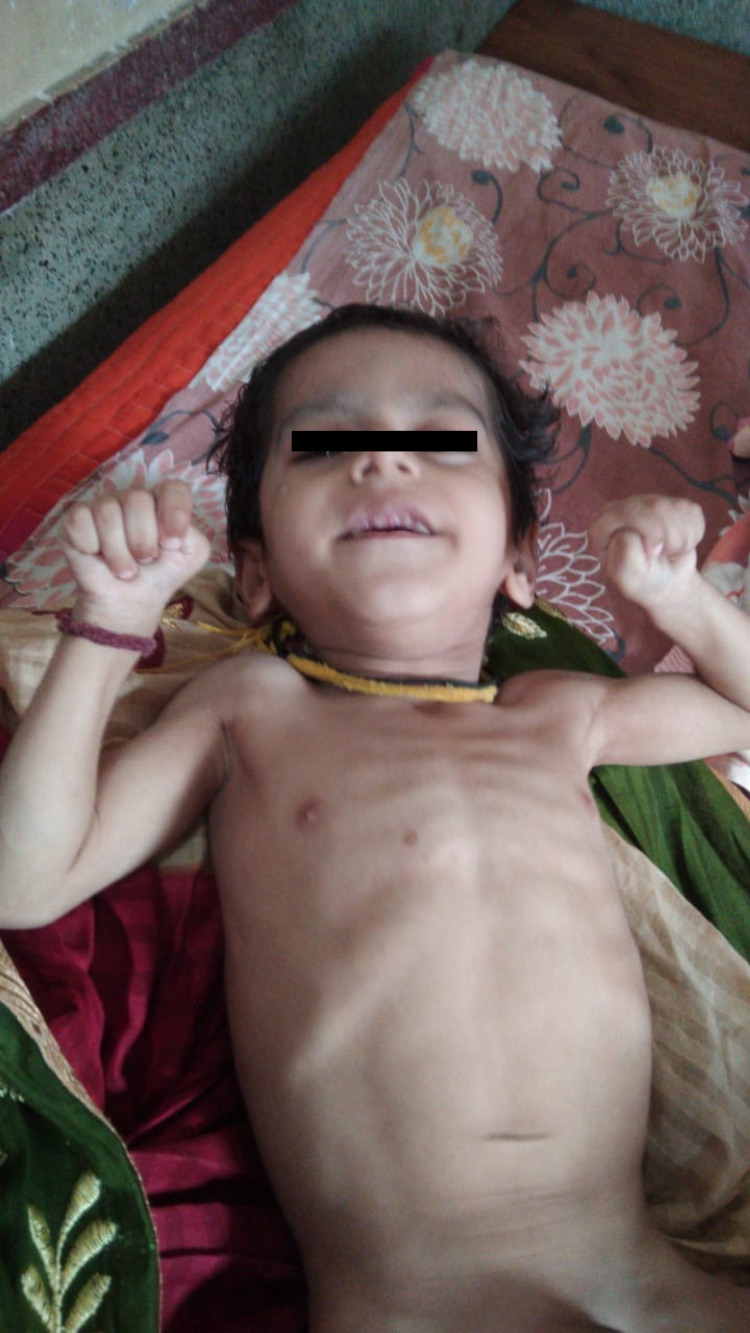
Failure to thrive and severe wasting

 A three-year-old boy presented with complaints of not achieving developmental milestones as per age and failure to gain weight. The child was first brought to medical attention at the age of nine months when parents felt that he was not developing like other children his age. There were also some concerns of neuro-regression according to parents as the child had lost the previously acquired milestone (achieved late, around one and half years of age) of sitting without support. At the time of presentation (three years of age), the child could not sit or stand independently and was completely dependent on the caregiver for all activities of daily living. There was no history of seizures. Other associated problems were difficulty in feeding the child, as he had frequent vomiting and suffered from chronic constipation. Birth history revealed that this child was born of a non-consanguineous marriage, born full-term by vaginal delivery with face presentation, with a birth weight of 2.75 kg. There were no ante-natal or peri-natal issues and the baby didn’t require neonatal intensive care unit (NICU) admission. There was no family history of any neurological illness and the patient had an older, six-year-old, female sibling who was well and normal. On examination, the child looked severely malnourished with anthropometry showing a weight of 5.7 kg and a head circumference of 42 cm (Figure 1). Previous clinical records revealed that the weight of the child was 4.6 kg at nine months and 6.1 kg at 22 months with the head circumference (HC) being 39 cm at nine months and 40.5 cm at 11 months. All values of weight and HC were below the 3 Z-score, showing severe failure to thrive and microcephaly.

**Figure 2:**
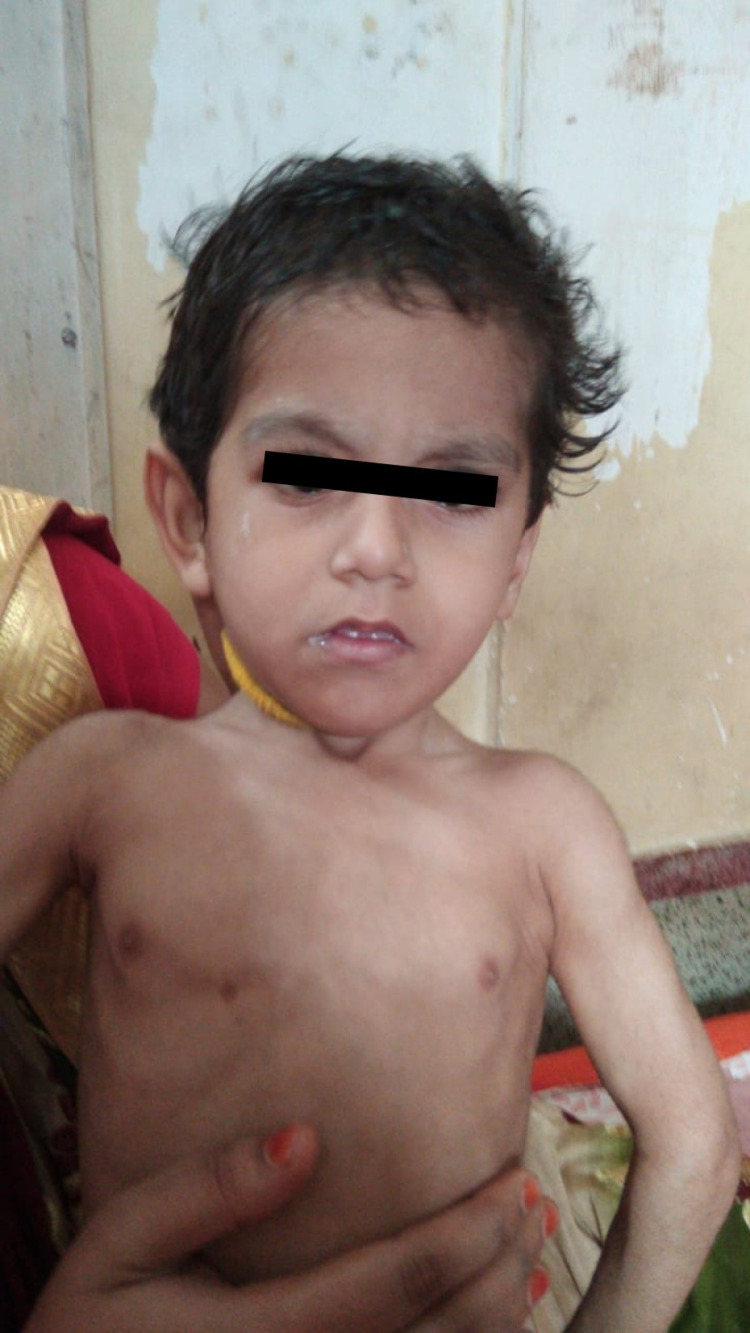
Dysmorphic facies

**Figure 3:**
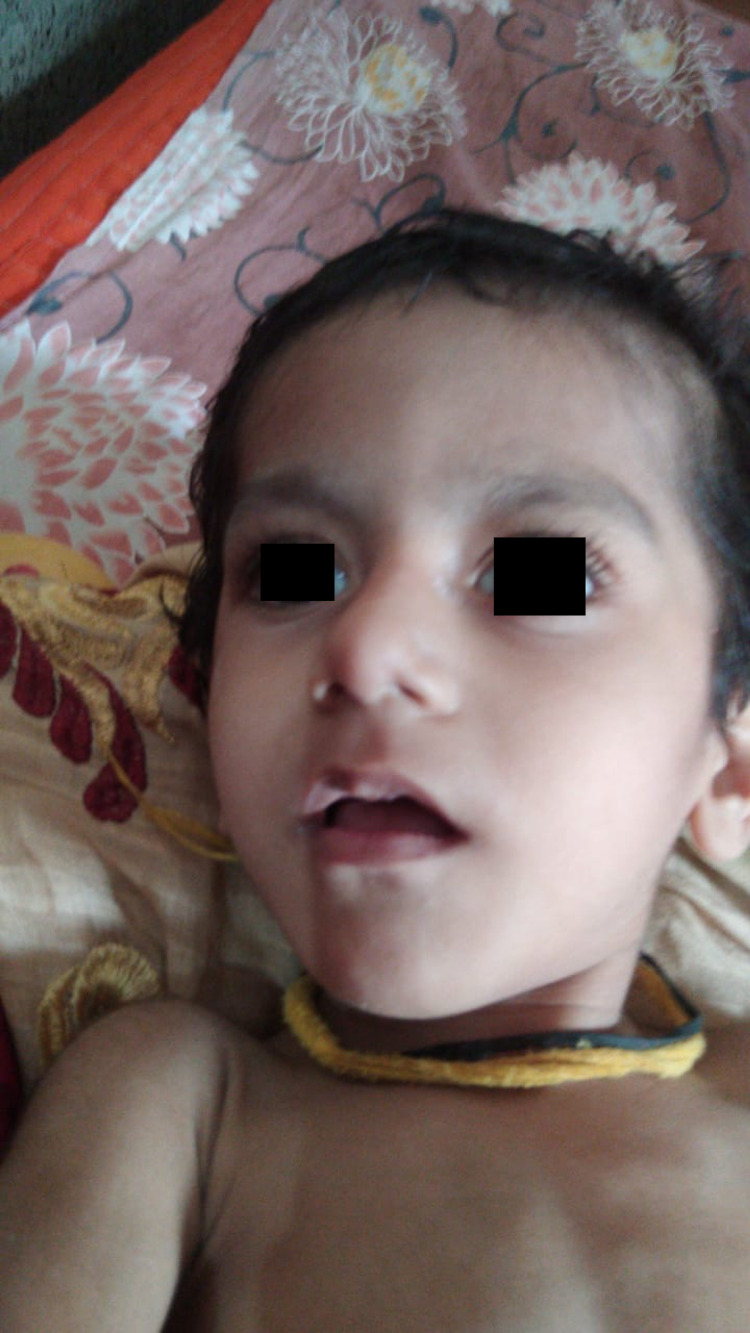
Bulbous nose tip, thin vermilion of the upper lip, mouth open

The child had dysmorphic features in the form of triangular facies, prominent eyebrows and eyelashes, bulbous nose tip, thin vermilion of the upper lip, prominent ears, and his mouth was always open (Figures 2-3).

**Figure 4:**
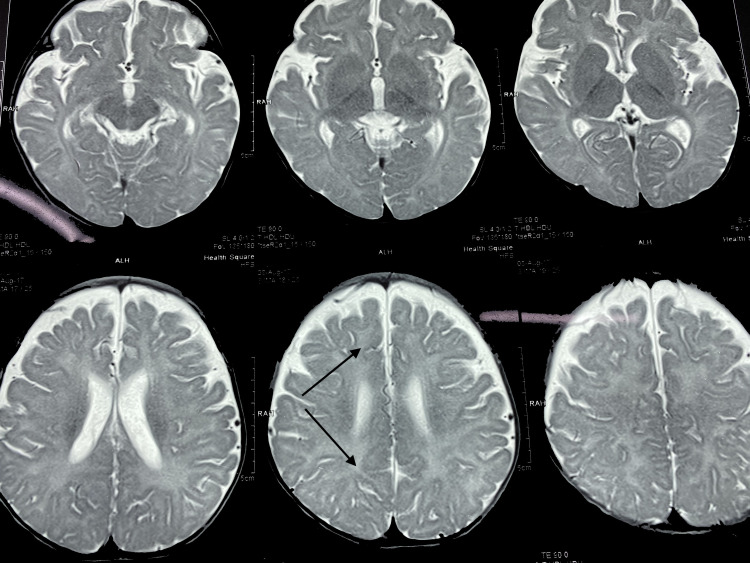
Hypomyelination of deep and subcortical white matter on T2-weighted images

**Figure 5:**
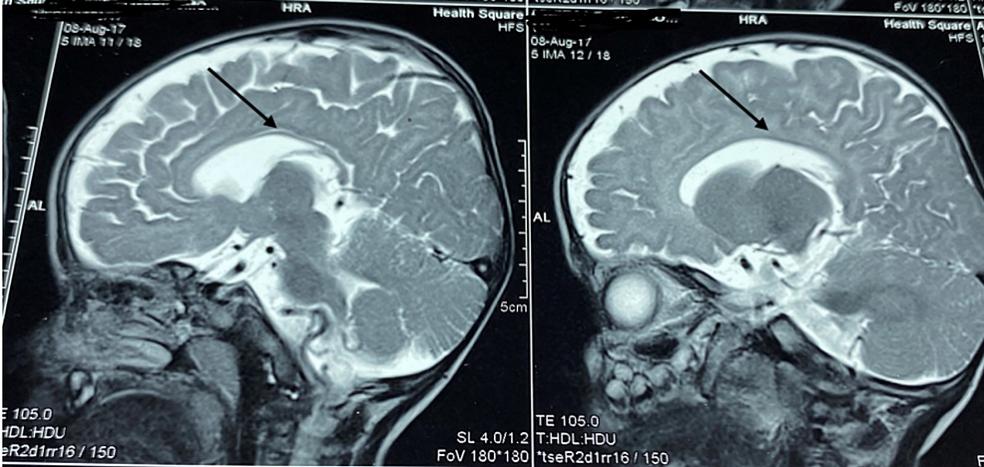
Thin corpus callosum

**Figure 6:**
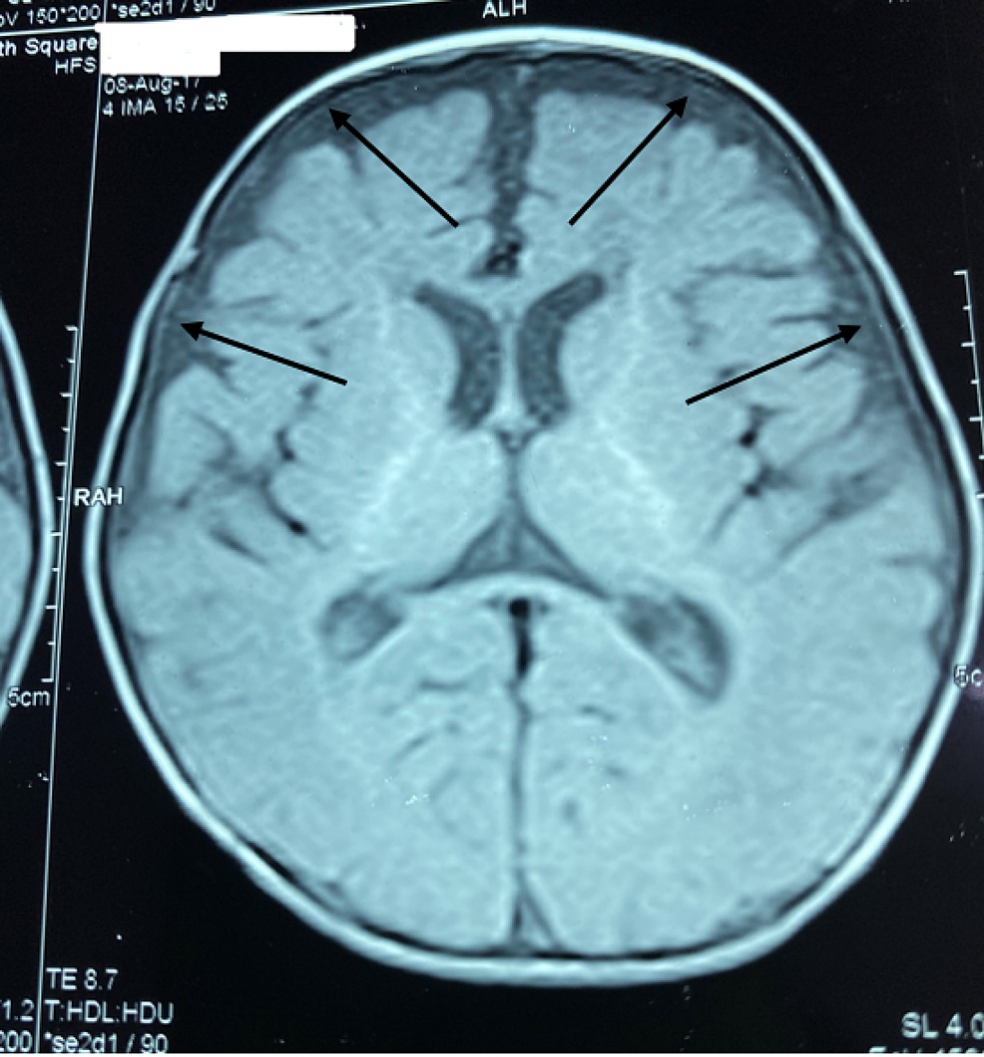
Increased extra-axial spaces suggesting cerebral atrophy

No fasciculations were noted in the tongue. There was axial hypotonia with partial head lag, appendicular hypertonia, and spasticity of limbs. Deep tendon reflexes were brisk, the plantars were bilateral extensor, and the range of motion of the hips and knees was limited. The child’s functional status was classified as Gross Motor Function Classification System (GMFCS) level V. The fundus was normal. Extraocular movements were full, pupils were equally reactive, and no facial asymmetry was noted. Basic blood tests including complete blood count, liver function test, renal function test, thyroid profile, calcium, plasma ammonia, and lactate were normal. Tests for inborn errors of metabolism in the form of blood tandem mass spectrometry (TMS) and urine gas chromatography-mass spectrometry (GCMS) were normal. MRI done at nine months of age showed hypomyelination of deep and subcortical white matter, including U fibers appearing hyperintense in T2WI and isointense in T1WI. The corpus callosum was thin (Figures 4-5). Bilateral internal capsules showed normal myelination for age with normal signals in both T1WI and T2WI. Cortical sulci, including Sylvian fissures, was diffusely prominent with dilated extra-axial spaces suggesting cerebral atrophy (Figure 6). One more finding that was noted was an extra-axial arachnoid cyst in the right anterior temporal region, which had a signal identical to cerebrospinal fluid (CSF) in all imaging sequences. Brainstem, cerebellum, and deep gray matter nuclei were normal.

**Figure 7:**
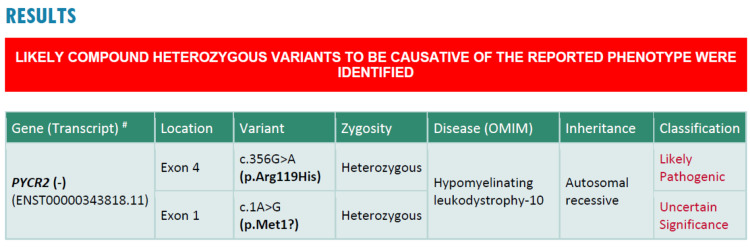
Genetic test (clinical exome sequencing) report showing a compound heterozygous mutation in the PYCR2 gene

As MRI was done at nine months of age, a repeat MRI was planned at the time of current consultation to look for interval change but parents were keen to go for the next step, which was the genetic test. A genetic test, in the form of clinical exome sequencing, was done, which showed a compound heterozygous mutation in the PYCR2 (pyrroline-5-carboxylate reductase 2) gene, a heterozygous missense variation in exon 4 (p.Arg119His), and another heterozygous start-loss variation in exon 1 (p.Met1). Hence, on combining the clinical phenotype with the genetic test result, a final diagnosis of PYCR2 gene-related hypomyelination and microcephaly syndrome (also known as hypomyelinating leukodystrophy 10) was established (Figure 7). Parents were explained about the diagnosis, course, and progression of the disorder, as well as the poor outcome. The patient was given pharmacotherapy for spasticity, and physiotherapy and occupational therapy were recommended. In view of the progressive neurological condition, severe wasting, and feeding difficulties, a percutaneous endoscopic gastrostomy (PEG) tube feeding was recommended but parents were not keen on it. As genetic counseling was not available in our center, parents were advised to seek genetic counseling from another center.

## Discussion

The enzyme, pyrroline-5-carboxylate reductase 2, encoded by the PYCR2 gene, is one of the three homologous enzymes that catalyzes the last step of proline synthesis. The other two enzymes are PYCR1 and PYCRL. A defect in proline synthesis causes major impairments in the form of intellectual disability, hypomyelination, skin and joint hyperelasticity, osteopenia, and cataracts [5-6]. Mutations in PYCR1 and PYCR2 cause very distinct clinical features. Homozygous mutations in PYCR1 have been identified in patients presenting with cutis laxa and progeroid features [7-8]. Nakayama et al., in 2015, described four children from two consanguineous families with microcephaly and HLD having PYCR2 variants. The study demonstrated that PYCR2-deficient cells showed increased apoptosis under oxidative stress and, therefore, postnatal microcephaly in affected children was most likely due to increased cell death in the CNS [4,9]. They also demonstrated that PYCR2 deficiency weakens mitochondrial membrane potentials, and, hence, the process of myelination, being an energy-dependent process, gets impaired, resulting in hypomyelination [4,10-11].

Zaki et al., in 2016, described 14 patients from 11 consanguineous families with homozygous PYCR2 mutations and similar clinical presentation [12]. The key clinical features noted by them were early presentation within the first year of life with profound neurodevelopmental delay, progressive microcephaly, characteristic triangular facies with large ears, upturned bulbous nose, malar hypoplasia, and motor signs like axial hypotonia, appendicular hypertonia, spasticity, and failure to thrive. Another study by Meng et al., in 2016, described five children from three different families with homozygous variants in the PYCR2 gene with progressive microcephaly and HLD [2]. A total of three out of five children were from non-consanguineous marriage unlike the cases of Nakayama et al. and Zaki et al. where consanguinity was present in all.

An extensive review of literature from various databases did not reveal any more cases of PYCR2-associated HLD beyond the fore mentioned 23 cases from three studies. There were no reports of similar cases from India and the authors believe that this is the first case of PYCR2-related HLD from India. Our case also presented early at nine months of age with global developmental delay, motor signs, head lag, and microcephaly. His birth weight was normal, and the neonatal period was uneventful. He appeared to grow normally in the first two to four months but thereafter a developmental delay became evident and neurologic signs became progressive, and he developed severe wasting, spasticity, and microcephaly. The MRI done at nine months of age showed hypomyelination and a repeat was planned to confirm the previous findings and see the interval change but parents were anxious and wanted to take the genetic test (clinical exome sequencing) that was planned after MRI. Clinical exome sequencing revealed a compound heterozygous mutation in the PYCR2 gene while in the literature so far, all the cases reported had a homozygous mutation in the PYCR2 gene. The clinical phenotype matched the genotype and helped in establishing the diagnosis.

Prognosis in hypomyelinating leukodystrophy-10 appears poor with most of the children succumbing in the first decade of life. Treatment is mostly supportive and requires a multidisciplinary team to address various issues like spasticity, feeding difficulties, constipation, mobility, nutritional deficiencies, etc. associated with this debilitating disorder. A genetic test should be recommended for the parents to see if they are heterozygous carriers of the mutated gene and, if present, would require genetic counseling before subsequent pregnancies to prevent a recurrence.

## Conclusions

There is a wide range of etiologies for debilitating neurologic disorders, which have common, overlapping clinical presentations. Causes of global developmental delay, microcephaly, upper motor neuron signs, and failure to thrive can be varied. A detailed history, thorough clinical examination, neuroimaging, and relevant laboratory investigations help in narrowing down the differential diagnosis. Advances in the field of genetics, growing awareness, and the gradual availability of genetic tests in smaller cities of developing countries is helping in the better evaluation of complex neurological disorders and establishing a complete diagnosis. A precise diagnosis helps in outlining the course, treatment (if available), and prognosis of the disease to parents and plays a vital role in planning future pregnancies.
